# Characterization of biocarbon-source recovery and microbial community shifts from waste activated sludge by conditioning with cornstover: Assessment of cellulosic compositions

**DOI:** 10.1038/srep42887

**Published:** 2017-02-17

**Authors:** Kaili Wen, Aijuan Zhou, Jiaguang Zhang, Zhihong Liu, Guoying Wang, Wenzong Liu, Aijie Wang, Xiuping Yue

**Affiliations:** 1 College of Environmental Science and Engineering, Taiyuan University of Technology, Taiyuan, China; 2 State Key Laboratory Breeding Base of Coal Science and Technology Co-founded by Shanxi Province and the Ministry of Science and Technology, Taiyuan University of Technology, Taiyuan, China; 3 College of Architecture and Civil Engineering, Taiyuan University of Technology, Taiyuan, China; 4 Research Center for Eco-Environmental Sciences, Chinese Academy of Sciences, Beijing, China; 5 State Key Laboratory of Urban Water Resource and Environment, Harbin Institute of Technology (SKLUWRE, HIT), Harbin, China

## Abstract

Most studies on the production of volatile fatty acids (VFAs) from waste activated sludge (WAS) digestion have focused on operating conditions, pretreatments and characteristic adjustments. Conditioning by extra carbon sources (ECS), normally added in a solid form, has been reported to be an efficient approach. However, this has caused considerable waste of monomeric sugars in the hydrolysate. In this study, the effects of two added forms (pretreated straw (S) and hydrolyzed liquid (L)) of cornstover (CS) on WAS acidification were investigated. To obtain different cellulosic compositions of CS, low-thermal or autoclaved assisted alkaline (TA or AA) pretreatments were conducted. The results showed that AA-L test achieved the highest VFAs value (653 mg COD/g VSS), followed by AA-S (613 mg COD/g VSS). These values were 12% and 28% higher, respectively, than that obtained in the TA-L and TA-S tests. Meanwhile, higher percentages of acetic acid were observed after AA pretreatment (~62% versus ~53% in TA). The added forms of CS played an important role in structuring the innate microbial community in the WAS, as shown by high-throughput sequencing and canonical correspondence analysis. The findings obtained in this work may provide a scientific basis for the potential implementation of co-digesting WAS with ECS simultaneously obtaining energy and high value-added products.

In China, the amount of waste activated sludge (WAS) from wastewater treatment plants (WWTPs) has been growing rapidly in recent years. Its treatment and disposal to avoid polluting to the environment is becoming a popular topic. In 2013, 6.25 million tons of dry sludge was produced in China and this value is rapidly increasing at an annual growth of 13%^1^. On this basis, more cost-efficient and benign alternatives and strategies are needed to better handle and utilize WAS. Anaerobic digestion (AD) is acknowledged as a cost-effective method to recover renewable resources (e.g., methane, hydrogen, etc.)^2^,^3^,^4^. However, many researchers have paid more attention to produce volatile fatty acids (VFAs) from WAS because of their long digestion times and complexities of their purifyication for methane production. As a class of high added-value chemical materials, VFAs are promising substrates for many bioprocesses, e.g., biopolymer production^5^, bioenergy generation^6^,^7^ and biological nutrient removal (BNR)^8^. Moreover, their composition has also been demonstrated to be closely related with many bioprocesses. For instance, a mixture of acetic acid (HAc) and propionic acid (HPr) was required to promote the growth of phosphorous accumulating organisms over other competing organisms^9^. For co-polymer production, mixtures of 98% 3-hydroxybutyrate (3HB) + 2% 3-hydroxyvalerate (3HV) and 7% 3HB + 83% 3HV + 10% 3-hydroxy-2-methylvalerate (3H2 MV) were produced by using HAc and HPr as the sole feedstocks, respectively^5^.

Until now, most of the reported studies on VFAs production from WAS digestion have focused on the influence of the operating conditions and pretreatment methods. However, the relatively low carbon to nitrogen (C/N) ratio in WAS (i.e., inefficiency of the carbohydrate) was confirmed to limit the VFAs yields by previous studies^10^,^11^. To achieve a higher VFAs yield, a variety of extra carbon sources (ECS) (e.g., municipal solid wastes, agricultural residues, paper wastes, food wastes, etc.) were applied to adjust the C/N ratio from approximately 7 to 15~70, which was the most suitable nutrient proportion for AD^12^. Jia *et al*.^13^ chose perennial ryegrass as a carbon substrate and concluded that the maximal VFAs yield was 369 g COD/kg TS at a C/N of 20/1, which was over 12 times that obtained at a C/N of 7/1 (only WAS)^13^. Hong and Wu (2010) observed that the VFA concentration achieved high yields (29099 mg/L) from dewatered excess sludge conditioned by food wastes with a proportion of 88%^14^. Feng *et al*.^15^ found the maximal VFAs yield (520.1 mg COD/g VSS) occurred during the co-digestion of rice and WAS, which was 3-fold more than that in WAS only (150.2 mg COD/g VSS)^15^. All these studies proved that characteristics adjustment by the addition of ECS could promote VFAs accumulation from WAS digestion.

Cornstover (CS) is one of the most abundant agricultural residues in China. Currently, conventional CS disposal routes are mainly land applications, incineration, landfills, silage or industrial utilization^16^. CS consists of high amounts of cellulose, hemicellulose and a relatively low amount of lignin and, is an ideal ECS feedstock for the conversion of WAS to VFAs. However, due to the crystalline structure of cellulose and the non-water soluble nature of lignin, cellulosic materials are not capable of undergoing fermentation without pretreatment and hydrolysis. Our previous study showed that a 69% increase in the VFAs yield was obtained by co-digesting WAS with low-thermal assisted alkaline (TA) pretreated CS as compared with that produced by sludge alone^17^. Meanwhile, a positive correlation existed between the VFAs yield and the content of cellulose and hemicellulose in the feedstock^18^. However, CS pretreatment causes not only the removal of lignin but also the solubilization of portions of cellulose and hemicellulose into monomeric sugars (such as glucose and xylose) in an aqueous phase (hydrolysate). Cao *et al*.^19^ showed that 27.4% hemicellulose and 10.2% cellulose were solubilized into the liquid phase after lime pretreatment of CS; the corresponding soluble substances were increased by 32.3%^19^. Thus, it can be thus speculated that considerable cellulosic materials are wasted if they are simply pretreated in the solid form. To gain a basic understanding of the suitable conditioning methods for WAS acidification, it is crucial to investigate the effects of the added forms of the ECS on VFAs production from WAS and related functional microbial community structures.

Based on the above considerations, the objective of this study was to evaluate the feasibility of improving WAS acidification by co-digesting with two added forms of CS (straw (S) and hydrolysate liquid (L)) by means of process assessment associated to the microbial community response analysis. Previous studies have shown that the temperature of pretreatment is a crucial factor for reducing the crystallinity of lignocellulosic biomass^20^. Thus, to investigate the effect of the cellulosic composition, in either the S or L forms of CS, on VFAs production from WAS, two commonly used combined pretreatments, TA and autoclaved assisted alkaline (AA), were examined. Hydrolysis, acidification and methanogenesis of particulate organic matter in the mixture feedstocks were also monitored. Furthermore, we also examined the microbial community structure, using high-throughput pyrosequencing of the 16S rRNA gene, which can provide important information to better understand the microbial response mechanism to the different WAS and CS co-digestion systems^21^. Correlations between the environmental variables and microbial populations were assessed using canonical correspondence analysis (CCA).

## Material and Methods

### Characteristics and pretreatment procedure of substrates

WAS was collected from the Jinzhong municipal wastewater treatment plant (Taiyuan City, China) and concentrated by settling at 4 °C for 24 h. Prior to being used as feed, the WAS was screened with a 1 mm sieve to remove impurities to prevent clogging problems. Ultrasonic pretreatment of the WAS was performed with a 28 + 40 kHz ultrasonicator; other operation parameters were documented in a previous publication^17^. The main characteristics (average value plus standard deviation of three tests) of the concentrated and pretreated WAS are displayed in Table 1.

The CS used in this study was collected at Taiyuan City, Shanxi Province, China. The chopped CS was dried in an oven at 70 °C to a constant weight. Then, it was milled to 2–10 mm before storage at room temperature prior to testing. The volatile solids content was 0.84 g volatile solids/g dry solids. TA pretreatment of CS was performed in a thermostatic water bath at 85 °C with a solid-liquid ratio of 1:10 (g dry weight to mL). The NaOH concentration was 2% (w/w) and the residence time was 1 h. AA pretreatment was performed in an autoclave at 121 °C for 15 min. The solid-liquid ratio and NaOH concentration was the same as the TA pretreatment. The main compositions of raw and pretreated CS straw (dry basis) and liquid are shown in Table 2. The straw was separated by centrifugation (10000 rpm, 10 min) (Sigma 3K30, Germany) and then dried at 70 °C to a constant weight and milled to 1–2 mm. The straws and liquids of pretreated CS were added for balancing the C/N ratio during WAS fermentation.

### Experimental setup and operation

Batch experiments were carried out in fifteen batch reactors. These reactors, with 300 mL of the mixed substrates each, were divided into five groups (three reactors were replicates for each group). The feedstock for group I was only the ultrasonic pretreated WAS (hereinafter referred to as the control test). Based our previous study, the CS proportion was set as 35% for the four WAS and CS co-digesting groups^17^. Two groups were fed with the mixtures of the ultrasonic pretreated WAS and AA pretreated CS liquid/straw (hereinafter referred to as the AA-L and AA-S tests, respectively). Two groups were fed with the mixtures of ultrasonic pretreated WAS and TA pretreated CS liquid/straw (hereinafter referred to as the TA-L and TA-S tests, respectively). The added amounts of CS liquid were in accordance with the corresponding straw tests. The main characteristics of the substrates were displayed in Table 1. After flushing with nitrogen gas to remove oxygen, all bottles were capped, sealed, and stirred in an air-bath shaker (100 rpm) at 35 ± 1 °C.

### DNA extraction and pyrosequencing

Before DNA extraction, sludge samples were centrifuged at 8000 g to remove the supernatant. DNA was extracted from sludge sediments of three replicate reactors using an EZNA^®^ Soil DNA kit (Omega Bio-Tek, Inc., Norcross, GA, USA) and then pooled together. Amplicon libraries were constructed for pyrosequencing using the bacterial fused primers 341 F and 805 R for the V3-V4 region of the 16S rRNA gene. To achieve sample multiplexing during pyrosequencing, barcodes were incorporated between the 454 adaptor and forward primer. The procedure for the polymerase chain reaction (PCR) reactions was performed in our previous study^18^. After being purified and quantified, the PCR amplicon was used for pyrosequencing on an Illumina MiSeq. The raw sequences were deposited in the NCBI Short Read Archive database with the accession no. SRR3602034. The adapters, barcodes, and primers in all raw sequences were trimmed to minimize the effects of random sequencing errors. Sequences shorter than 350 bp or containing any ambiguous base cells, were removed.

The remaining sequences were clustered into operational taxonomic units (OTUs), using a 97% identity threshold (3% dissimilarity level). Rarefaction curves were generated and alpha diversity measurements, including Shannon indexes ((http://www.mothur.org/wiki/Shannon) and Simpson indexes (http://www.mothur.org/wiki/Simpson), were calculated for each sample. Beta diversity was calculated using the distance matrices generated using the phylogenetic-based method UniFrac^22^ and then visualized using principal coordinates analysis (PCoA). Canonical correspondence analyses (CCA) were conducted by Canoco 4.5 to examine correlations between characteristic genera and the environmental and performance measurements, including methane, pH, VFAs, HAc, HPr, soluble proteins and carbohydrates concentrations. The relative abundance of 16 characteristic bacterial was used in the CCA analysis. A Venn diagram showing shared and unique OTUs was used to depict the similarities and differences between the five communities.

### Analytical methods

Sludge samples were centrifuged at 10000 rpm after anaerobic fermentation, then filtered through a 0.45 μm cellulose nitrate membrane filter and finally stored at 4 °C, prior to analysis. The determination of TSS, VSS, SCOD, TCOD, carbohydrates, and proteins was performed as previously described^23^,^24^. One liter Cali-5-Bond™ gas-sampling bags were used to collect the biogas produced. The total volume of gas was measured using a glass syringe (50 mL). The gas composition was analyzed using a gas chromatograph (GC) (4890D, Agilent), equipped with a thermal conductivity detector (TCD). The cellulose, hemicelluloses and lignin content of the CS were measured using a Fiber Analyzer (ANKOM, USA). The xylose, arabinose and glucose concentrations in the CS hydrolysate were measured using high performance liquid chromatography (HPLC) (model e2695, Waters Co., Milford, MA). Another Agilent 7890 GC, equipped with a flame ionization detector (FID), was utilized to analyze the composition of the VFAs. The VFAs production was calculated as the sum of the measured acetic (HAc), propionic (HPr), n-butyric (n-HBu), iso-butyric (iso-HBu), n-valeric (n-HVa) and iso-valeric (iso-HVa) acids. The COD conversion factors are 1.50 g COD/g protein (assumed as (C_4_H_6.1_O_1.2_N)x), 1.06 g COD/g carbohydrate (assumed as C_6_H_12_O_6_), 1.07 g COD/g HAc, 1.51 g COD/g HPr, 1.82 g COD/g HBu, and 2.04 g COD/g HVa.

## Results and Discussion

### VFAs production and composition

**Figure 1:**
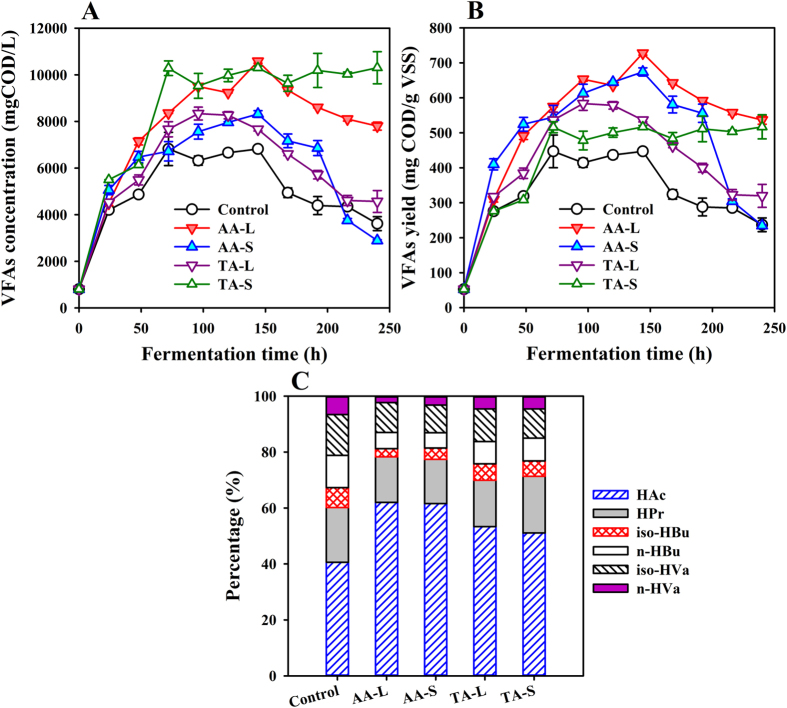
Effect of CS addition forms and pretreatment methods on VFAs concentration (**A**), yield (**B**) and composition (**C**) from WAS co-digestion (Note: the error bars represent the standard deviation).

The total VFAs production during anaerobic co-digestion of WAS and CS with the feedstock proportions of 65%: 35% (VSS_WAS_:VSS_CS_) is shown in Fig. 1A. It was seen that the VFAs concentration sharply increased in all reactors from 96 h onward, and no significant VFA increase was observed after 96 h of fermentation time. In this sense, the optimal fermentation time was 96 h for the VFAs production. At that time, the maximum VFAs concentration were, respectively, 9498 ± 83, 7563 ± 321, 8338 ± 276, 9527 ± 534 mg COD/L with AA-L, AA-S, TA-S and TA-L as the carbon additions. The concentration for sludge alone (i.e., the control test) was only 6320 ± 196 mg COD/L because of the un-balanced C/N fermentation environment. Apparently, VFAs production was successfully generated by co-digesting WAS with the two forms of CS, which has also been proven to be an effective method in many cases^13^,^15^,^25^. Meanwhile, the VFAs production was closely related with the additional CS forms and compositions (resulting from different pretreatments). Interestingly, the time-course curve showed that the sequence of the VFAs concentration for the AA conditioning tests was AA-L > AA-S. Comparatively, a different observation was made for the TA pretreatment with the sequence TA-S > TA-L. That is, when CS was pretreated with different methods, although the same added form of CS was applied for balancing the WAS nutrients, adverse effects on the VFAs production were observed. Apparently, there indeed existed a synergistic effect between the WAS and CS when they were fermented together. As far as VFAs yield was concerned, the AA-L and AA-S addition caused higher values (653 and 613 mg VFAs-COD/g VSS at 96 h), which were, respectively, 12% and 28% higher than those obtained in the TA-L and TA-S tests (Fig. 1B). That is, conditioning with AA pretreated CS in liquid form had a better effect on VFAs recovery from WAS.

The individual VFA production and percentage accounting for the total VFAs at 96 h are shown in Fig. 1C, when the total VFAs had reached a plateau in most of the reactors. According to the composition analysis, the top three (individual) VFAs produced were HAc, HPr and iso-HVa in both of the AA and TA co-digesting tests no matter what the added form was, but they varied to different extents. This was also consistent with the individual WAS digestion (41%, 20% and 15% for HAc, HPr and iso-HVa, respectively) (Fig. 1C). Similarly, this was also confirmed by the research undertaken by previous studies during the co-digestion of *Agaricus bisporus* substrates and WAS^26^. Figure 1C also showed that the CS composition indeed affected the distribution of the individual VFAs, whereas the added form of CS had little effect on it. Just as shown, the percentage of iso-HVa decreased from 15% in the control test to ~10% and ~11% in AA and TA tests, respectively. Similarly, the percentages of HBu (both n-HBu and iso-HBu) and n-HVa were all decreased. Oppositely, the corresponding percentage of HAc was obviously increased (from 41% in the control to ~53% and ~62% in TA and AA tests, respectively). Seemingly, the addition of carbohydrate substrate boosted the β-oxidation process, producing more low-molecular-weight VFAs. This phenomenon was also observed in previous studies^17^,^27^. In addition, conditioning with AA-pretreated CS contributed more to the distribution of HAc than conditioning with TA-pretreated CS, in which the proportion was enhanced by approximately 10%.

### Time-courses of soluble organics changes and methane production

**Figure 2:**
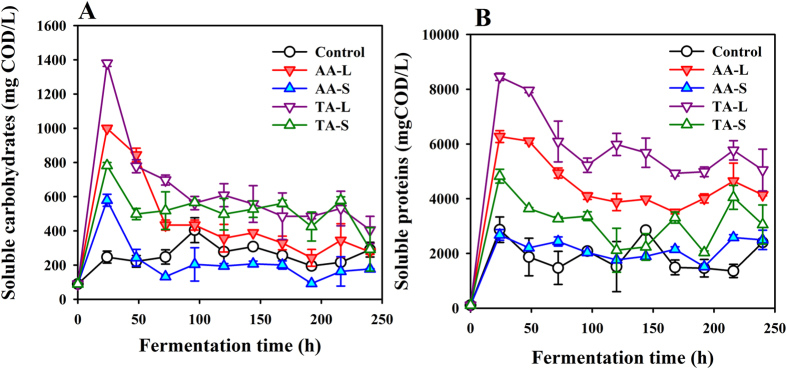
Time-course profiles of soluble carbohydrates (**A**) and soluble proteins (**B**) (Note: the error bars represent the standard deviation).

Given that the distribution and production of VFAs were very different in the four groups, it was necessary to explore the underlying mechanisms. It was well known that proteins and carbohydrates were the main constituents of the WAS (the amount of lipids was very small, so it was not taken into consideration). The variation trends of the two kinds of organic matter were detected during a 240 h fermentation time. Figure 2 shows that the soluble proteins and carbohydrates sharply decreased from 96 h onward and then fluctuated at a relatively low level in all tests. The consumption of proteins and carbohydrates increased in all co-digesting tests, which can better express the relationship between the organic matter and VFAs produced. The accumulation of the soluble organics depended on its rate of production and consumption. Supposing that the production rate of the soluble organics equaled that of consumption, the specific consumed proteins were, respectively, 2853, 1642, 3230 and 2297 mg COD/L in the AA-L, AA-S, TA-L and TA-S tests, which were 2.7-, 1.6-, 3.1- and 2.2-fold of those in the control test (1045 mg COD/L). Apparently, the consumption of proteins was increased by the addition of the carbohydrate substrate (CS). In fact, the similar trend for the carbohydrates was also observed, corroborating the positive function of extra carbohydrate addition on the VFAs production from WAS, which was in accordance with other studies^15^,^17^. In addition, the hydrolysates, including monomeric sugars derived from parts of the cellulose and hemicellulose, in the CS pretreated liquid can better stimulate the degradation of proteins, no matter what pretreated CSs were utilized. This was consistent with the higher production rate of the VFAs in the liquid tests.

The pH value is of great importance to the production of VFAs because most acidogens can only survive in the pH range of 4.0–8.5^28^. Just as shown in Fig. S1A,B, the pH value rapidly reduced from 96 h onward in all tests because of the production of the VFAs. In the TA-S, AA-S, TA-L and the control tests, the pH values were gradually increased with the action of methanogens, such as transformation of VFAs to biogas and alkali production in the form of carbon dioxide, ammonia and carbonate^29^. When VFAs were formed in the system, methane was produced immediately but increased slowly from 96 h onward because of the low concentration of VFAs and the unstable pH value (Fig. S1C,D). Then, the methane production increased sharply when the pH value tended to remain neutral. The obtained data fitted the linear growth model (*Y*_CH4_ = *constant + kt*), and the corresponding production rate constants (*k*) were calculated. The specific methane yield slightly increased from 12.5 ± 0.9 mL/g VSS (*k* = 0.0621 h^−1^, R^2^ = 0.9539) in the control sample, to 16.0 ± 0.8 (*k* = 0.0563 h^−1^, R^2^ = 0.8178) and 16.1 ± 0.2 mL/g VSS (*k* = 0.0729 h^−1^, R^2^ = 0.8507) in the TA-S and TA-L samples, respectively, and further to 22.6 ± 2.1 mL/g VSS (*k* = 0.0872 h^−1^, R^2^ = 0.7797) in the AA-S sample, while it was inhibited in the AA-L sample after 240 h of fermentation time. It is known that the pH range of 6.5–7.2 is optimal for most methanogenic bacteria^29^. In the AA-L, the pH value decreased gradually to an approximately constant value (5.9) from a relatively high value of 8.7, which was not conducive to methanogenesis and prevented the consumption of the produced VFAs for methane formation^30^. This explained why the methane production was almost zero and less VFAs were consumed by methanogens. Moreover, more lignin in the AA-pretreated CS was removed (12.8% versus 8.7% in the TA pretreated CS) and then solubilized into liquid, which thereby introduced an inhibitory effect on the methanogenic micro-organisms in the AA-L. The corresponding microbial community analysis is listed later, corroborating the effect of the added forms and compositions of CS on the methanogens.

### Overall analysis of pyrosequencing

**Figure 3:**
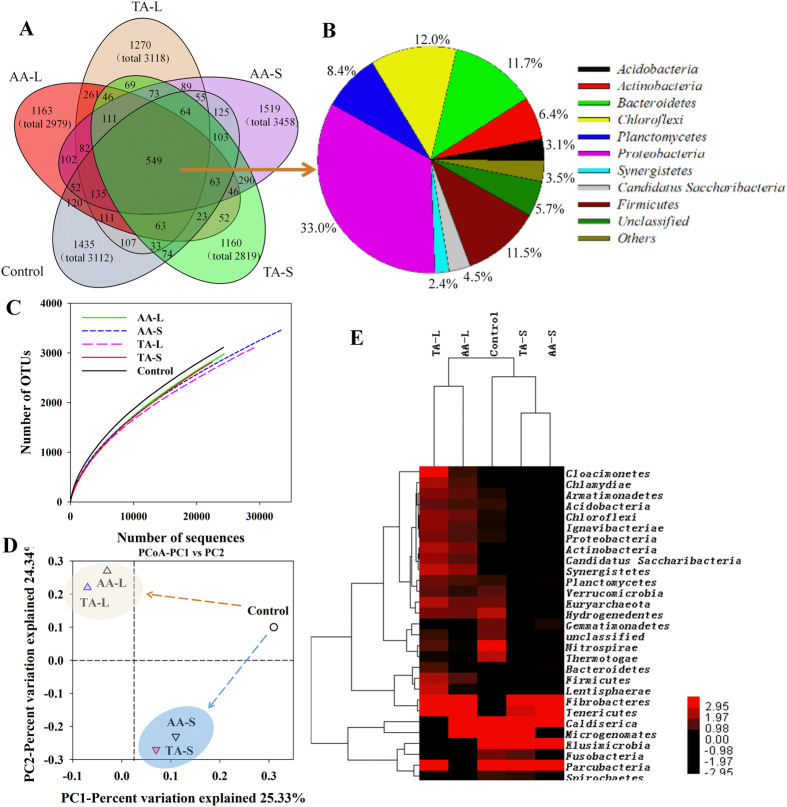
Overlap of the five bacterial communities based on OTU (3% distance) (**A**). The shared OTUs were analyzed at phylum level (**B**). Rarefaction curves (**C**) and principal component analysis (**D**) of bacterial communities from WAS and WAS-CS based on pyrosequencing of 16 S rRNA gene. Hierarchical cluster analysis (**E**).

Microorganisms in the anaerobic systems played important roles in the production of VFAs and methane during fermentation of the WAS and CS. To understand the mechanisms of digestion, high-throughput pyrosequencing was conducted to evaluate the microbial diversity and distribution. Just as shown in Fig. 3A, the total number of operational taxonomic units (OTUs) in the five bacteria samples was 10569, in which 549 OTUs (5.2% of the total OTUs) were shared by all samples. The shared OTUs were mainly grouped into four phyla: *Proteobacteria* (33.0%), *Bacteroidetes* (11.7%), *Chloroflexi* (12.0%) and *Firmicutes* (11.5%) (Fig. 3B). The number of OTUs shared by the AA-L and TA-L samples was 261, while 290 were shared by the AA-S and TA-S samples. However, the AA-L and AA-S samples shared less OTUs (102), and the same result was observed with the TA-L and TA-S samples (69 OTUs). There were in total 6547 OTUs (61.9% of the total OTUs) that were unique to the five samples. The rarefaction curves for all libraries displayed shapes indicative of effective sampling of the community diversity. However, new bacterial phylotypes continued to emerge even after 22000 reads sampling with pyrosequencing (Fig. 3C).

The microbial diversities of the evolving communities were assessed based on α-diversity (Table S1). Based on the Shannon indexes, the control sample had a relatively higher diversity (6.29) than that of other four samples. The bacterial communities in the AA-L and AA-S (5.86 and 5.63) were slightly more diverse than those in the TA-L and TA-S (5.79 and 5.29). In addition, the diversity of the L-conditioning tests was higher than that of the S-conditioning tests. Taking the Simpson indexes into consideration, the value of the control (0.007) was much lower than that of the other samples. The two kinds of indexes illustrated that the microbial diversity of the four WAS and CS co-digesting samples was substantially reduced compared to the control sample. That is, the proportions of functional microorganisms were elevated simultaneously. The similarity of the microbiome was calculated and examined by β-diversity. According to the PCoA of the classified OTUs in the five samples, generated from unweighted UniFrac, there was a big difference between the communities of the control sample and the other four co-digesting samples (Fig. 3D). The principal components 1 and 2 accounted for 25.33% and 24.34%, respectively, of the total community variation. The AA-L and TA-L had relatively similar communities that were obviously distinct from those of the AA-S and TA-S. A hierarchical clustering analysis was also conducted in order to further illustrate the distribution and the similarity of the microbial communities (Fig. 3E). There were three clusters. The TA-S and AA-S were clustered together. The TA-L microbial community was highly similar to that of the AA-L, while they were all very distinct from that of the control sample, suggesting clear distinctions in the community structure between different sludge digestion systems despite the fact that the same initial source of WAS microbial consortia was shared. These results show that particular bacteria were selectively enriched in the WAS digestion systems by conditioning with CS, and the different added forms had an obvious effect on the community structures.

### Microbial community distribution and diversity analysis

**Figure 4:**
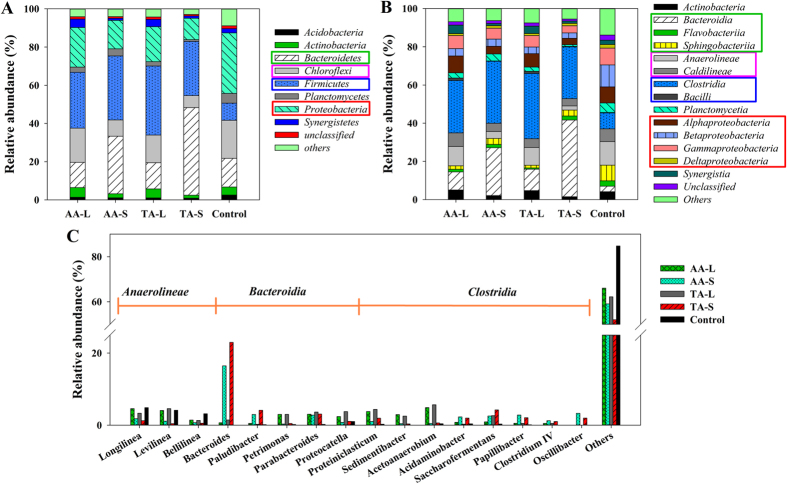
Taxonomic classification of pyrosequences from bacterial communities of five samples at the phyla (**A**), class (**B**) and genus (**C**) levels.

To investigate the diversity and distribution of the microbial community, bacteria at the phyla, class and genus level were included in a phylogenetic analysis of the 16 S rRNA gene sequences. Clear changes were observed in the microbial community structures during WAS co-digestion with CS in the different added forms and compositions (Fig. 4). The four dominant phyla in five samples were *Bacteroidetes, Chloroflexi, Firmicures* and *Proteobacteria* (Fig. 4A), which were common fermentation phyla observed in many studies^18^,^31^,^32^. The total proportions of dominant phyla were in the range of 75.7–91.7% for the five samples. The two most abundant phyla in the control sample, *Proteobacteria* and *Chloroflexi* (31.7% and 19.9%), decreased in the four co-digesting samples. The proportion of *Proteobacteria* (31.7%) in the control sample decreased to a relatively lower value by S-conditioning (14.9% and 11.2% for the AA-S and TA-S) than that by L-conditioning (20.7% and 18.3% for AA-L and TA-L). In addition, the same phenomenon for *Chloroflexi* was also observed. However, *Firmicutes* increased to a high level after co-digestion with CS, which was closely related with hydrolysis and acidification. *Bacteroidetes* (15.0% for the control), as an exception, decreased slightly by S-conditioning (13.2% and 13.7% for AA and TA) but increased sharply by L-conditioning (30.0% and 45.8% for AA and TA).

Fourteen major classes included the majority of the sequences at the class level (Fig. 4B). Among them, *Clostridia* (phylum *Firmicutes*) and *Bacteroidia* (phylum *Bacteroidetes*) were the dominant ones. *Clostridia*, accounting for more than 90% of *Firmicutes*, have been reported to be capable of releasing hydrolases to utilize various organics and produce VFAs under anaerobic conditions^15^,^33^. *Clostridia* were evidently enhanced in all co-digesting samples (from 8.3% in the control to 27.1–34.1%). *Bacteroidia* also took up the largest proportion for co-digesting tests, especially for TA-S and AA-S (40.1% and 25.0%), which was merely 2.9% for Control. *Bacteroidia* were one of the few types of bacteria resistant to the extreme pH conditions and have been reported to play a critical role in sludge reduction^34^.

Further investigation on the genus level provided more detailed information about the microbial communities (Fig. 4C and Table S2). Four genera of *Bacteroidetes (Bacteroides, Paludibacter, Parabacteroides* and *Petrimonas*) were identified; the proportions were all increased in the four co-digesting samples. *Bacteroides* contributed significantly to VFAs accumulation because most of them could produce HAc, HPr, formate and succinic acids^35^ with the proportions of 16.5% and 23.0% for AA-S and TA-S, respectively. *Paludibacter* has been commonly associated as a strictly anaerobic, HPr - producing bacterium^36^, and it was enriched to ~3.6% by S-conditioning. Oppositely, the other two *Bacteroidetes* reached the highest abundance by L-conditioning. As a saccharolytic genera (~3.4% by L-conditioning), *Parabacteroides* was closely related with the degradation of polysaccharides and accompanied by the production of HAc and succinate^37^. Likewise, similar to *Parabacteroides, Petrimonas*, which can utilize various sugars and produce HPr and HAc as primary products^36^,^38^, peaked to ~3.0% by L-conditioning versus ~0.4% by S-conditioning. *Proteocatella* and *Sedimentibacter* (belonging to the class *Clostridia*) also increased in the AA-L and TA-L. Both of them are proteolytic genera, closely related with HAc and HBu production^39^,^40^. *Proteiniclasticum*, which cannot utilize carbohydrate but soya peptone, tryptone and amino acids^41^, was mainly detected by L-conditioning (~4.1%) as well. Its major fermentation products were HAc, HPr and iso-HBu. Moreover, the proportions of *Proteocatella* and *Proteiniclasticum* were slightly higher in the TA pretreated samples than those in the AA samples (Table S2). The other dominant genera for L-conditioning tests were *Ethanoligenens* (class *Clostridia*, ~2.3%) and *Acetoanaerobium* (class *Bacilli*, ~5.3%), which can, respectively, ferment several kinds of mono-, di- and oligosaccharides to form HAc^42^ and produce HAc from H_2_ and CO_2_^43^. It should be noted that *Acidaminobacter, Saccharofermentans, Papillibacter, Clostridium IV* and *Oscillibacter* (belonging to class *Clostrdia*) had totally different variance trends with the genera just mentioned above, being abundant only in the AA-S and TA-S. *Clostridium IV* was involved in the VFAs production and has been reported to produce cellulases, lipases, proteases, and other extracellular enzymes^44^. *Oscillibacter*, a valerate producer, is closely associated with the metabolism of various substrates^45^.

### Correlation between the environmental variables and microbial populations

**Figure 5:**
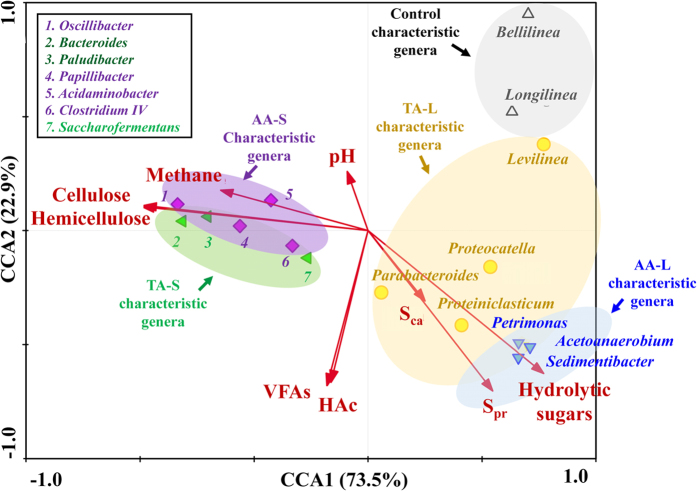
Canonical correspondence analysis (CCA) between enriched genera and environmental variables (VFAs, methane, pH, soluble proteins and carbohydrtaes).

To further figure out the plausible correlation between different conditioning conditions (CS added forms and compositions), characteristic genera and various environmental and performance measurements, including pH values and methane, VFAs, HAc, hydrolytic sugars, cellulose, hemicellulose, soluble proteins (S_pr_) and carbohydrates (S_ca_) concentrations were taken into consideration by CCA (Fig. 5). Based on the assumption that the WAS digestion process is most likely driven by predominant bacteria, we performed a CCA using 16 characteristic bacteria. The contents of the S_pr_, S_ca_ and hydrolytic sugars were proven to be positively correlated with the first canonical axis (explaining 73.5% of the variance of the genera distribution), and the contents of methane, VFAs, HAc, cellulose, hemicellulose, and the pH values showed negative interrelations. For axis 2 (explaining 22.9% variance), only methane, pH, cellulose and hemicellulose showed good positive correlations. The detailed information is shown in Table S3.

The length of an arrow-line indicates the strength of the relationship between the environmental variable and microbial community. As indicated, hydrolytic sugars, celluloses and hemicelluloses were strongly linked to the microbial community according to the length of the vector, followed by the S_ca_ values. The characteristic genera in the S_conditioning tests were more likely to be enriched by feedstocks with high contents of cellulose and hemicellulose, which had a highly positive correlation with the methane production (AA-S: *Obcillibacter, Papillibacter, Acidaminobacter, Clostridium IV* and TA-S: *Bacteroides, Paludibacter, Saccharofermentants*). When the content of the hydrolytic sugars increased in the feedstock, the characteristic genera in the L_conditioning tests were enriched, which all showed good positive correlations with VFAs, HAc, S_pr_ and S_ca_ (AA-L: *Petrimonas, Acetoanaerobium* and *Sedimentibacter* and TA-L: *Proteocatella, Proteiniclasticum* and *Parabacteroides*). Moreover, in view of the CCA results, the three characteristic genera in the AA-L were also closely related with the HAc. This was in accordance with the above analysis results in the *VFAs production and composition* section. As indicated, the S_pr_ had a relatively positive correlation with the genera distribution. The reason of this phenomenon may be that the addition of a carbohydrate substrate boosted the release of proteins from WAS. In addition, the intersection angle between the S_pr_ and HAc was slightly greater than that of the S_ca_, indicating that the S_ca_ was more related to HAc production than S_pr_. This was verified by the yield coefficients of HAc from monosaccharides and amino acids (i.e., ƒ_ac,su_ and ƒ_ac,aa_), which were 0.41 versus 0.40, respectively^46^. Meanwhile, the changes of the S_ca_ and S_pr_ concentrations were related to the VFA production (including HAc), indicating that the efficiency of the WAS hydrolysis played an important role for the subsequent acidification process. In addition, cellulose, hemicellulose and pH were closely related to the methane production, which was indicated by the intersection angles. This result was consistent with the above discussions (in the *Time-courses of soluble organics changes and methane production* section). In this sense, the CCA results suggested that the added forms of the external carbon source may play an important role in structuring the innate microbial community in the WAS, and the relationship between the community structure and the measured variables may reveal the whole CS and WAS co-digestion process.

### Significance and potential implementation

**Figure 6:**
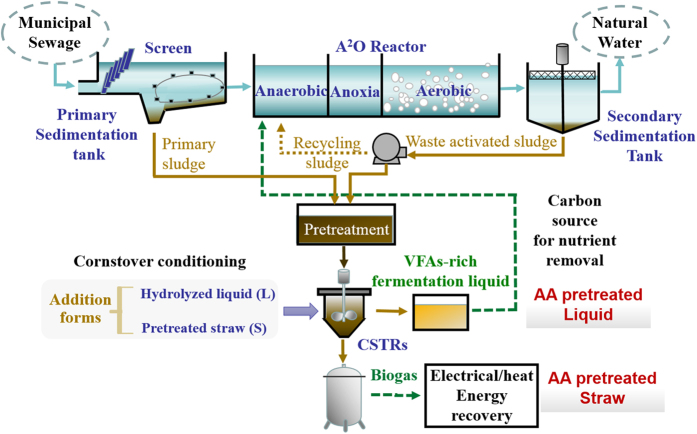
Schematic diagram of an enhanced concept applied in a WWTP with the WAS digestion conditioning with different CS addition forms.

The phenomenon that conditioning with different added forms of the extra carbon source affects the WAS digestion, as explored in this work, sheds light on a new approach to promote resource utilization from anaerobic digestion. This study reveals details of how different added forms and compositions of extra carbon sources affect the WAS digestion efficiency by means of process assessment associated to the microbial community response analysis. More importantly, the findings obtained in this study might have crucial implications for the operation of WWTPs. With the growing worldwide energy crisis, it has been estimated that WWTPs are increasingly recognized as energy producers rather than conventional waste removers by reducing the embedded energy and practicing energy recovery from WAS. In this sense, there is an ongoing paradigm shift of the operation of WWTPs from pollutant removal to energy recovery. Figure 6 illustrates an enhanced “carbon source recovery - nutrient removal - energy recovery” concept with WAS digestion, which includes conditioning with different added forms and compositions of CS applied in a WWTP. It is known that the fermentative VFAs from WAS have drawn more and more attention because they can act as not only the external carbon sources employed for biological nutrient (nitrogen and phosphorus) removal^9^,^47^, but also the key intermediates involved in the production of methane during anaerobic digestion. Thus, when using WAS as an energy source in a WWTP, VFAs production should be maximized. As stated above, the AA-L test gained the highest VFAs value (653 mg COD/g VSS), followed by the AA-S (613 mg COD/g VSS), which were, respectively, 12% and 28% higher than those obtained in the TA-L and TA-S tests. Previous studies indicated that the spectrum of acids in the WAS fermentation liquid is ideal for supporting BNR. Among the six VFAs, HAc was regarded as the favorite substrate for BNR processes^48^. The percentage of HAc was significantly increased from 41% for WAS digestion alone to ~53% for TA pretreatment and further to ~62% for AA pretreatment. Some researchers also explored almost the same approach to produce HAc by co-digesting with other carbon-rich substrates. Jia *et al*.^13^ showed the HAc percentage increased from 28.9% (C/N 7.0, sludge alone) to 48.7% (C/N 26.0, WAS and perennial ryegrass). Morgan-Sagastume *et al*.^27^ showed that the HAc ratio to other acids was higher when fermenting the mixture of primary sludge and WAS^27^. Based on the above considerations for both production yield and composition, it is ideal to harvest VFAs as target products from WAS digestion conditioned with AA-L CS. In addition, WAS contains a significant amount of embedded energy, on the order of 20 MJ/kg of dry sludge^49^. More methane was produced in the AA-S test (22.6 ± 2.1 mL/g VSS, 10 d), which was higher than that produced in the other co-digestion and control tests. Thus, the proposed concept in Fig. 6 may be a practical way for WWTPs to co-digest sludge with extra carbon sources and gain energy and high value-added products simultaneously, by changing the added forms of the CS. The findings obtained in this work may provide a scientific basis for the implementation of this carbon recovery process from WAS digestion for the operation of WWTPs.

## Conclusion

This study observed the effects of CS conditioning, with different added forms and compositions on the performance of WAS acidification and suggested crucial implications for the operation of WWTPs. A comprehensive study to shed light on the underlying mechanism was undertaken using process assessment associated with the microbial community analysis. Autoclaved alkaline pretreated CS in liquid form (AA-L) exerted a positive influence on the VFAs production and composition (higher HAc proportion) from WAS. In addition, that in the solid addition form (AA-S) preferably stimulated methane production. Further investigation revealed that the microbial communities were significantly shifted in different WAS and CS co-digesting systems by high-throughput sequencing analysis. The findings obtained in this study might have crucial implications for the operation of WWTPs to co-digest sludge with extra carbon sources and to simultaneously obtain energy and high value-added products.

## Additional Information

**How to cite this article**: Wen, K. *et al*. Characterization of biocarbon-source recovery and microbial community shifts from waste activated sludge by conditioning with cornstover: Assessment of cellulosic compositions. *Sci. Rep.* **7**, 42887; doi: 10.1038/srep42887 (2017).

**Publisher's note:** Springer Nature remains neutral with regard to jurisdictional claims in published maps and institutional affiliations.

## Supplementary Material

Supporting Information

## Figures and Tables

**Table 1:** The main characteristics of the substrates.

Parameter	Concentrated WAS^a,b^	Pretreated WAS^a,b^	Autoclaved-alkaline		Thermal-alkaline	
			Liquid	Straw	Liquid	Straw
pH	6.68~6.90	6.62~6.76	6.62~6.76	6.62~6.76	9.39~9.51	7.71~7.88
TSS	24390 ± 1320	22930 ± 820	24510 ± 468	27880 ± 727	23040 ± 794	29690 ± 482
VSS	16160 ± 980	15260 ± 1140	14540 ± 577	12343 ± 372	14290 ± 760	19930 ± 985
SCOD	232 ± 27	4290 ± 192	13371 ± 811	7785 ± 838	7362 ± 1012	6897 ± 996
TCOD	25420 ± 641	26930 ± 314	19660 ± 1463	20843 ± 1672	28460 ± 1455	31580 ± 4198
VFAs (as COD)	69 ± 6	805 ± 17	2552 ± 997	1524 ± 572	596 ± 112	989 ± 37
Soluble carbohydrates (as COD)	89 ± 7.6	412 ± 22	1631 ± 82	603 ± 116	1499 ± 112	1012 ± 79
Soluble proteins (as COD)	110 ± 17.2	3130 ± 419	6950 ± 601	3675 ± 374	10930 ± 869	11525 ± 599

^a^All values are expressed in mg/L except pH.

^b^The error bars represent the standard deviation.

**Table 2:** Characteristics of raw and pretreated CSs.

		Raw CS	AA-pretreated CS	TA-pretreated CS
Straw (Residue)	Cellulose (%)	37.1 ± 1.2	47.7 ± 0.8	39.8 ± 1.1
	Hemicellulose (%)	28.4 ± 0.8	16.8 ± 1.2	15.7 ± 1.1
	Lignin (%)	18.5 ± 1.3	5.7 ± 0.7	9.8 ± 0.9
Liquid (Hydrolysate)	Glucose (g/L)	—	1.9 ± 0.3	2.8 ± 0.3
	Xylose (g/L)	—	4.9 ± 0.7	5.5 ± 0.6
	Arabinose (g/L)	—	0.7 ± 0.1	1.0 ± 0.2
