# Supplementary material for: Characterization of biocarbon-source recovery and microbial community shifts from waste activated sludge by conditioning with cornstover: Assessment of cellulosic compositions
Source: Sci Rep. 2017 Feb 17;7:42887. doi: 10.1038/srep42887 (PMC5314333; doi:10.1038/srep42887)
Supplement: Supporting Information [file srep42887-s1.doc]

**Supporting** **information for**

**Characterization of biocarbon-source recovery and microbial community shifts from waste activated sludge by conditioning with cornstover: Assessment of cellulosic compositions**

Kaili Wen, Aijuan Zhou ^*^, Jiaguang Zhang, Zhihong Liu, Guoying Wang, Wenzong Liu, Aijie Wang and Xiuping Yue ^*^

***Corresponding author.** E-mail: [zhouaijuan@tyut.edu.cn](mailto:zhouaijuan@tyut.edu.cn) (A. Zhou); [yuexiuping@tyut.edu.cn](mailto:yuexiuping@tyut.edu.cn) (X. Yue)

# Contents

Supporting tables (Table S1, S2, S3)

Supporting figures (Figure S1)

# Supporting tables

Table S1 Alpha diversity parameters

| Name   |  | | --- | |  | | Seq num*^1^ | OTU num*1 | Shannon-index | Simpson |
| --- | --- | --- | --- | --- | --- | --- |
| AA-L | 24349 | 2979 | 5.86 | 0.012 |
| AA-S | 33379 | 3458 | 5.63 | 0.029 |
| TA-L | 29309 | 3118 | 5.79 | 0.012 |
| TA-S | 22495 | 2819 | 5.29 | 0.050 |
| Control | 24256 | 3112 | 6.29 | 0.007 |

^*1^: “Seq num” indicated the sequence numbers obtained from the high-throughput sequencing analysis;

^*2^: “OTU num” indicated the classified OTU numbers obtained from the gene sequences with the identity of over 97 %.

Table S2. Genera (relative abundance >1% in at least one samples)

| **Genera** | **AA-L** | **AA-S** | **TA-L** | **TA-S** | **Control** |
| --- | --- | --- | --- | --- | --- |
| ***Bacteroides*** | **0.66** | **16.46** | **1.45** | **22.97** | **-** |
| *Mangroviflexus* | - | 0.35 | 0.01 | 6.65 | 0.05 |
| ***Paludibacter*** | **0.49** | **2.97** | **0.24** | **4.13** | **0.14** |
| ***Parabacteroides*** | **3.03** | **2.79** | **3.65** | **3.07** | **0.22** |
| ***Petrimonas*** | **2.99** | **0.31** | **2.98** | **0.45** | **0.19** |
| *Proteiniphilum* | 0.81 | 0.19 | 0.99 | 0.25 | 0.05 |
| *Sunxiuqinia* | 0.49 | 0.14 | 0.3 | 0.45 | 1.35 |
| *Azohydromonas* | 0.11 | 0.17 | 0.04 | 0.1 | 1.42 |
| *Nitrosospira* | 0.25 | 0.3 | 0.26 | 0.28 | 1.35 |
| *Rubrivivax* | 0.02 | 0.03 | - | 0.02 | 1.13 |
| *Thauera* | 0.38 | 0.11 | 0.17 | 0.14 | 1.66 |
| *Fulvimonas* | 0.94 | 1.66 | 0.93 | 0.92 | 1.79 |
| *Haliea* | - | 0.48 | - | 0.32 | 1.79 |
| *Steroidobacter* | 2.12 | 1.28 | 1.72 | 0.86 | 1.98 |
| *Sulfurovum* | 0.11 | 0.07 | 0.67 | 0.03 | 1.10 |
| *Hyphomicrobium* | 1.06 | 0.29 | 0.71 | 0.24 | 0.85 |
| *Bellilinea* | 1.42 | 0.7 | 1.33 | 0.55 | 3.16 |
| *Levilinea* | 4.07 | 1.12 | 4.59 | 0.41 | 4.13 |
| *Longilinea* | 4.58 | 1.79 | 3.31 | 1.24 | 4.86 |
| *Aciditerrimonas* | 1.84 | 0.65 | 1.46 | 0.56 | 0.92 |
| *Ilumatobacter* | 1.86 | 1.06 | 1.81 | 0.68 | 2.63 |
| *Caldilinea* | 1.07 | 0.32 | 0.52 | 0.32 | 0.87 |
| *Litorilinea* | 6.03 | 4.04 | 4.07 | 3.47 | 5.84 |
| ***Acetoanaerobium*** | **4.87** | **0.47** | **5.67** | **0.62** | **0.38** |
| ***Acidaminobacter*** | **0.79** | **2.30** | **0.28** | **1.95** | **0.36** |
| *Anaerovorax* | 1.13 | 0.74 | 1.05 | 0.65 | 0.67 |
| *Caloramator* | - | 4.29 | 0.01 | 0.74 | - |
| ***Clostridium IV*** | **0.48** | **1.23** | **0.48** | **1.01** | **0.07** |
| *Clostridium XlVa* | 0.11 | 1.73 | 0.08 | 1.65 | 0.01 |
| ***Ethanoligenens*** | **2.46** | **0.38** | **2.07** | **0.37** | **0.31** |
| *Fusibacter* | 0.18 | 0.46 | 0.2 | 0.4 | 1.97 |
| ***Papillibacter*** | **0.51** | **2.81** | **0.5** | **2.07** | **0.09** |
| ***Proteiniclasticum*** | **3.80** | **1.01** | **4.37** | **1.93** | **0.26** |
| ***Proteocatella*** | **2.39** | **0.75** | **3.76** | **1.07** | **1.02** |
| *Ruminococcus* | 0.02 | 0.42 | 0.01 | 1.18 | - |
| ***Saccharofermentans*** | **0.90** | **2.54** | **2.62** | **4.22** | **0.30** |
| ***Sedimentibacter*** | **2.92** | **0.41** | **2.51** | **0.33** | **0.04** |
| *Sporobacter* | 0.18 | 4.56 | 0.35 | 2.44 | 0.01 |
| *Syntrophomonas* | 0.95 | 0.03 | 2.18 | 0.02 | 0.31 |
| *Tissierella* | 0.85 | 0.05 | 2.01 | 0.05 | 0.04 |
| ***Oscillibacter*** | **0.06** | **3.28** | **0.07** | **1.95** | **0.02** |
| *Pirellula* | 0.74 | 0.99 | 0.59 | 0.24 | 1.38 |
| *Planctomyces* | 1.05 | 1.02 | 0.86 | 0.25 | 1.23 |
| *Ferruginibacter* | 0.71 | 0.81 | 0.48 | 0.59 | 2.18 |
| *Filimonas* | 0.15 | 0.50 | 0.09 | 0.47 | 1.25 |
| *Pseudosphingobacterium* | 0.14 | 0.44 | 0.11 | 0.62 | 1.76 |
| *Terrimonas* | 0.51 | 0.66 | 0.30 | 0.64 | 1.52 |
| *Cloacibacillus* | 3.6 | 0.82 | 3.10 | 0.94 | 1.47 |
| *Ignavibacterium* | 1.63 | 1.05 | 1.55 | 0.85 | 2.16 |
| *Nitrospira* | 0.06 | 0.04 | 0.06 | 0.03 | 1.36 |
| *Fluviicola* | - | 0.24 | - | 1.17 | 0.05 |
| *Lutaonella* | 0.99 | 0.19 | 0.30 | 0.29 | 1.15 |
| *Parachlamydia* | 0.93 | 0.85 | 1.20 | 0.38 | 1.05 |

Table S3 The eigenvalues of the first two canonical axes and their relationships with each environmental factor

|  | **Axis 1** | **Axis 2** |
| --- | --- | --- |
| Eigenvalues | 0.504 | 0.158 |
| Cumulative percentage variance | 73.5% | 96.4% |
| S_pr_ | 0.5471 | -0.7035 |
| S_ca_ | 0.2503 | -0.3085 |
| Methane | -0.6463 | 0.1777 |
| pH | -0.091 | 0.2592 |
| VFAs | -0.1624 | -0.6662 |
| HAc | -0.1801 | -0.6807 |
| Hydrolytic sugars | 0.7705 | -0.6266 |
| Cellulose | -0.9819 | 0.1104 |
| Hemicellulose | -0.9916 | 0.1046 |

# Supporting figure

**Figure S1** The variation of pH values during WAS and CS co-digestion (A: autoclaved-alkaline; B: thermal-alkaline); methane production from WAS (C: autoclaved-alkaline; D: thermal-alkaline) (Note: error bars represent standard deviation).
